# The Automatic Methods Group *Newsletter*

**DOI:** 10.1155/S1463924697000217

**Published:** 1997


Journal of Automatic Chemistry, Vol. 19, No. 5 (September–October 1997) pp. 183–185

AUTOMATIC METHODS GROUP

Analytical Division
ROYAL SOCIETY OF CHEMISTRY

### The Automatic Methods Group *Newsletter*

The AMG Newsletter is published as part of and in collaboration with the *Journal of Automatic Chemistry*. Further information on the *Journal of Automatic Chemistry* can be obtained from Taylor & Francis Ltd, Rankine Road, Basingstoke, Hampshire RG24 8PR or e-mail:info@tandf.co.uk; worldwide web home page: http:||tandf.co.uk.

### Conference Report

The 11th International LIMS Conference, which was held in the Netherlands from 3–5 June 1997, took the overall theme of information integration. The basics of laboratory computing were addressed in issues like system procurement, management, implementation and validation, and the programme also reflected the wider role of LIMS in the corporate environment, with a 500 sq m exhibition and a mix of plenary lectures, vendor workshops, attended posters and interactive breakout sessions. Topics included:

- *Human aspects of LIMS*: Implementation of a LIMS can be a traumatic and sometimes threatening experience for staff, yet, for a LIMS project to deliver its claimed benefits, it is essential to have the active support of staff. To improve understanding of the issues involved in the management of change, the programme featured Joseph Golden on 'Understanding the human dimension in LIMS projects'; and Gerry Newman and John Trigg on 'Why is big brother watching you?—Human resource issues in LIMS'.
- *Quality assurance and quality management*: A LIMS system documents the process of generating objective data and provides the audit trail to track results back to source documents. Thus audit of the LIMS itself, and of potential vendors, is critical. Papers at the meeting included Robert Megargle on 'Are there better ways to validate?'; Siri Segalstad on 'Vendor audits'; David Williamson on 'Re-engineering for regulatory compliance through LIMS'; and Allen Nakagawa on 'LIMS—a quality management tool for QA, validation and regulatory compliance.
- *LIMS and the internet*: Cyberspace is not a plaything for children; the internet was conceived for its power to connect people and the unrivalled business benefits which that process brings. Speakers on integration included: Peter Boogaard on 'The universal cyberlinks of tomorrow at your fingertips'; Reinhold Schaefer on 'LIMS in an internet/intranet environment—benefits of a new technology in the lab'; Tim Long on 'Development of an HTML-based intranet LIMS data delivery system'; and Jessie Paterson on 'Cost savings through the use of the internet'.
- *LIMS implementation*: Papers for those new to LIMS included: Diane Cameron and Russell Calow on 'LIMS: common design and installation problems and how to do it right'; Svendt Martin Fransen on 'Implementation of a "standard" LIMS'; Michael Wall on 'Life after LIMS at ALCON laboratories'; Birger Aune on 'A long and winding road—a history of LIMS implementation'; and Sandy Weinberg on 'How secure is your vendor? Evaluating the commitment and future of LIMS companies'.
- *Re-engineering/integration/the lab of the future*: The earliest LIMS implementations were around 30 years ago. As technology changes, one of the greatest challenges is to develop and re-engineer old systems. This was discussed by Ken Leiper; Randy Hice; Albrecht Kuhn and R. Roth; Peter Boogard; and Dale Seabrook.
- *Vendor sessions* included: Gerhard Stifter (HP, Germany) on 'Integration—LIMS, the automated laboratory, and production control'; JHM Souverin (BCO, Holland) on 'MOLIS and PARUNA: preparing for robotization'; John Boother (Autoscribe, UK) on 'LIMS configuration—a live demonstration'; and Stefaan van Haupt (Compex, Belgium) on 'LIMS within the framework of MES and EPR systems'.
- *Short courses* were run by Randy Collins on 'Re-engineering and workflow analysis'; Joseph Golden on 'A LIMS primer—an introduction to LIMS';

0142-0453/97 $12·00 © 1997 Taylor & Francis Ltd

Reinhold Schaefer on 'Implementation and integration of LIMS into a complex environment' and Siri Segalstad on 'LIMS validation'.

The meeting had 255 delegates and over 50 exhibitors. The next International LIMS conference will be held in June 1999, in Vienna.

**Alan McLelland** *Institute of Biochemistry, Royal Infirmary, Glasgow*

### AMG WORKSHOP Speciation and identification—Cost, value and credibility

*To be held on Tuesday 28 October 1997 at the Scientific Societies Lecture Theatre, London, UK*

In spite of the fact that the term 'speciation' still means different things to different people, speciation and identification are increasingly important issues facing the analytical scientist. This workshop programme has been structured to draw on the experiences of recognized practitioners over a broad spectrum of application areas and will provide an opportunity to understand the area-specific problems and definition of the term 'speciation'.

It will also provide an insight into common issues such as:

- availability of standards
- validation of methods
- data interpretation
- identification uncertainty and, where relevant, problems in quantitation of identified species.

The interactive nature of this workshop will also provide an excellent opportunity for delegates to exchange experiences and views, and provide a forum to discuss possible directions that future legislation may drive developments in this important area.

**Programme**

9.30 Registration and coffee

Chairman: Alex Williams (formerly the Government Chemist)

10.00 Speciation, academic novelty or real analytical challenge?
Professor Les Ebdon, University of Plymouth

10.45 Speciation in pharmaceuticals—the cost and the value
Howard Hill, Covance Laboratories

11.15 The value of atomic fluorescence spectroscopy in mercury, arsenic, selenium and antimony speciation studies—what can be achieved?
Warren T. Corns, Peter B. Stockwell and Louise Armstrong, PS Analytical Ltd

11.45 Discussion

12.00 Lunch

Chairman: Professor Peter B. Stockwell, PS Analytical Ltd

13.15 Identification of controlled substances in foods
Rob Massey, Food Science Laboratory, CEFAS

13.35 Interpreting the information—quantifying the certainty of identification
Bill Hardcastle, LGC

14.15 Problems encountered during the development of a method for the speciation of mercury and methylmercury by HPLC coupled to ICP-MS
Christopher Harrington and Tim Catterick, LGC

14.45 Discussion

14.55 Tea

15.15 Structured discussion, chaired by Ken Leiper

15.45 Where next?
Professor Les Ebdon

**Registration fees** RSC members £100, Non-members £140, and Students and retired members £60. A small number of student bursaries are available, please contact Dr Narayanaswamy for details. Payments should be made in pounds sterling by cheque or money order made payable to Automatic Methods Group RSC-AD. Payment by BACS accepted to Midland Bank, sort code 40-30-24, account No. 91244671.

For further information contact Dr R. Narayanaswamy, DIAS, UMIST, PO Box 88, Manchester, M60 1QD. Tel: 0161 200 4891/4885; Fax: 0161 200 4911; e-mail: ramaier.narayanaswamy@umist.ac.uk.

### Meetings: Preliminary notices

### Workshop: making the most of technology transfer—the academic industrial interface

*Preliminary Notice of a Meeting organized jointly by the Automatic Methods Group and the Analytical Division of the Royal Society of Chemistry to be held on Wednesday 25th February 1998 at The Scientific Societies Lecture Theatre, New Burlington Place, London WI, UK*

In an increasingly value-conscious culture, analytical scientists are being driven to find novel means of developing cost-effective solutions to problems. The inability to address these issues in-house through lack of resource or skill bases has provided the stimulus to seek external input in many organizations: This is now occuring at a level which would not have been considered a decade ago. In a relatively static employment environment, an increasing number of organizations are actively extending team working to include academia. Far from being the problem that might be anticipated, it is becoming increasingly apparent that successfully planned and managed projects are providing the opportunity not only for the development of successful methodology, but are also increasing the level of understanding of mutual needs. Interestingly, the lasting benefit lies in the opportunity such projects provide to develop and transfer skills rather than technology.

This programme will be of interest to all managers—whether academic or industrially-based—as well as to laboratory scientists and their customer base. It will cover all aspects of project management in joint ventures and will detail the availability of government sponsorship.

### Programme

10:30 Registration and coffee
11:25 Chairman's introduction—Professor D. Betteridge
11:35 The industrialist's view—What do we expect from the academic?
D White, BP Research, Sunbury
12:00 Lunch (provided)
13:00 The teaching company scheme—What opportunities does it offer analytical science?
Professor R. Burns, Plymouth Teaching Company Centre
13:25 The academic's view—Meeting the demands of industrial programmes ensuring deliveries at a sensible time scale
Professor L. C. Ebdon, University of Plymouth
13:55 A small company's perspective on technology transfer—'As ye sow so shall ye reap!'
Professor P. B. Stockwell, PS Analytical
14:20 Tea
14:50 The role of government
L. Davies, Link
15:15 Open discussion session—chaired by Ken Leiper
16:00 Close

Further details, and a registration form, can be obtained from the Hon. Secretary of the Automatic Methods Group: Derrick G. Porter, Willowfold, Fir Tree Lane, West Chiltington, West Sussex RH20 2RA. Tel: 01798 81 2383; e-mail: dgporter@argonet.co.uk.

